# Editorial: Women's Professional Sport: Understanding Distinctiveness

**DOI:** 10.3389/fspor.2021.806247

**Published:** 2021-12-10

**Authors:** Ann Pegoraro, Tracy Taylor

**Affiliations:** ^1^Gordon S. Lang School of Business and Economics, University of Guelph, Guelph, ON, Canada; ^2^College of Business and Law, RMIT University, Melbourne, VIC, Australia

**Keywords:** women, gender equity, professionalization, women's sport, sport careers

The accelerated growth of women's professional and semi-professional sport leagues and competitions has become a global phenomenon. While opportunities for women to forge careers as professional athletes have existed for decades in individual sports such as golf and tennis; the same pathways in team sports have historically been limited to a handful of sports [e.g., basketball; handball; football (soccer)] and within a limited geography (e.g., USA; Europe). As more sports recognise their growing societal contribution and consumer potential, there has been a relative explosion of new or expanded leagues (e.g., Athletes United, FA Women's Super League). This increase in opportunities for women athletes has led to an increasing professionalism of women's sport and expanded career options. However, corresponding scholarship into the personal, organisational, and societal aspects of women's professional sport is still in its infancy (Taylor et al., 2019). Research to date suggests that women in professional sport display many forms of distinctiveness, from their training/coaching regimes through to cultural and structural considerations. For this Research Topic, professional sport is delimited as any sport that provides continuous paid employment and the opportunity to pursue this employment as a career.

In recent history, women's professional sport has had its ups and downs as the sporting world has produced a number of women's sport competitions that have been launched with great hope only to then collapse. This was characterised by a period of well-publicised league “failures” in women's professional team sport in North America (Micelotta et al., 2018), notably associated with its franchise model. Outside North America, a common approach has been for national sport governing bodies to provide the impetus for the creation and support (resourcing) of women's leagues. Newer league offerings are still in the early phases of their product lifecycle, where most are focused on building their brand and fan base with the ultimate goal of achieving independent commercial viability (Mumcu et al., 2019). This growing momentum has been tempered to some extent by the impact of Covid-19 implications on women's competitions. However, some preliminary analysis indicates that women's professional leagues were not as negatively impacted as was expected, and in fact many saw increased viewership when the pandemic kept fans in their homes.

It was this moment offered by the global pandemic that led Lebel et al. to engage a wide range of stakeholders in women's sport to better understand the state of the women's sport landscape. In their article, the authors take us through various stakeholders' responses, demonstrating both similarities across the respondents but also that one's occupational lens (e.g., coach, administrator, professor) and one's age impacts how one frame the current state of women's sport, with younger respondents seeing the influence of women athletes in driving change while older respondents spoke more about legislation's (e.g., Title IX) role in making change. The authors leave us with a call to action to rethink and unlearn to essentially think beyond traditional structures.

Gender discrimination still plays a role in limiting participation, even in some of the countries where gender inequality is less severe. Meier et al., in their article examine the impact of gender (in)equality on country participation in international athletics utilising a unique dataset of season's bests results. Their findings indicate that gender inequality exists and that while women's participation in athletics has made considerable progress in the past two decades there is still a need for better targeted and resourced development programs aimed at increasing participation in less gender equal countries. The results from this study indicate that some nations are yet to realise the benefits of gender equality in areas such as women's participation in the labour force in regard to the socio-economic status of women, which have been empirically linked to success and participation in international women's elite sports.

Cosentino et al. article explores the theme of women's prospects in leadership roles in professional sport in Canada. While women have made strides in sport participation and in gaining entry level positions in the sport industry, their representation in leadership roles remains limited. The study captured the experiences of women in senior leadership positions in Canadian professional sport properties and suggestions proffered for women to successfully attain such roles. The authors offer recommendations for greater gender equality and inclusion in leadership positions.

Culvin and Bowes contribution examines the gender-specific needs of women in professional women's football in England, with a focus on maternity policy. They discuss the choices that women make about motherhood, their sport career, and the implications thereof. The professional women footballers interviewed offer insights about policy designs, career transitions, behavioural, and body choices. The authors highlight inadequacies of maternity policy in current contracts and provide suggestions that could enhance the career prospects of professional women footballers who become mothers.

In summary, this Research Topic provides much needed insights into women's professional sport, through research that highlights female-specific distinctiveness and provides empirical substantiation that can be used for evidence-based decision making and creating positive change.

Initiatives to improve access, inclusion and gender equality are more likely to be effective if they are guided by evidence. The papers in this Research Topic add to the growing body of knowledge in women's professional sport. The authors address issues that can inform evidence-based policies in regard to women's professional sport careers. Additionally, we hope that these contributions will drive researchers to further tackle the complexities that women face in building and advancing professional sport careers.

## Author Contributions

All authors listed have made a substantial, direct, and intellectual contribution to the work and approved it for publication.

## Conflict of Interest

The authors declare that the research was conducted in the absence of any commercial or financial relationships that could be construed as a potential conflict of interest.

## Publisher's Note

All claims expressed in this article are solely those of the authors and do not necessarily represent those of their affiliated organizations, or those of the publisher, the editors and the reviewers. Any product that may be evaluated in this article, or claim that may be made by its manufacturer, is not guaranteed or endorsed by the publisher.

## References

[B1] Micelotta E. Washington M. Docekalova I. (2018). Industry Gender Imprinting and New Venture Creation: The Liabilities of Womens Leagues in the Sports Industry. Entrep. Theory Pract. 42, 94–128. 10.1177/1042258717732778

[B2] Mumcu C. (2019). Business analytics in women's professional sports, in Handbook of the Business of Women's Sport, eds N. Lough, and A. Geurin (New York, NY: Routledge), 239–251.

[B3] Taylor T. Donna O'Connor D. Hanlon C. (2019). Contestation, disruption and legitimization in women's rugby league. Sport Soc. 23, 315–334. 10.1080/17430437.2019.1631803

